# Study of congenital Morgagnian cataracts in Holstein calves

**DOI:** 10.1371/journal.pone.0226823

**Published:** 2019-12-26

**Authors:** Marina Braun, Ann-Kathrin Struck, Sina Reinartz, Maike Heppelmann, Jürgen Rehage, Johanna Corinna Eule, Malgorzata Ciurkiewicz, Andreas Beineke, Julia Metzger, Ottmar Distl

**Affiliations:** 1 Institute for Animal Breeding and Genetics, University of Veterinary Medicine Hannover, Hannover, Germany; 2 Clinic for Cattle, University of Veterinary Medicine Hannover, Hannover, Germany; 3 Small Animal Clinic, Faculty of Veterinary Medicine, Freie Universität Berlin, Berlin, Germany; 4 Institute of Pathology, University of Veterinary Medicine Hannover, Hannover, Germany; University of Illinois, UNITED STATES

## Abstract

Cataracts are focal to diffuse opacities of the eye lens causing impaired vision or complete blindness. For bilateral congenital cataracts in Red Holsteins a perfectly cosegregating mutation within the *CPAMD8* gene (*CPAMD8*:g.5995966C>T) has been reported. We genotyped the *CPAMD8*:g.5995966C>T variant in Holstein calves affected by congenital bilateral congenital cataracts, their unaffected relatives and randomly selected herd mates. Ophthalmological examinations were performed in all affected individuals to confirm a congenital cataract. Whole genome sequencing was employed to screen variants in candidate genes for the Morgagnian cataract phenotype. In the present study, 3/35 cases were confirmed as homozygous mutated and 6/14 obligate carriers. Further 7/46 unaffected animals related with these cases were heterozygous mutated for the *CPAMD8*:g.5995966C>T variant. However 32 cases with a congenital cataract showed the wild type for the *CPAMD8* variant. We did not identify variants in the candidate genes *CPAMD8* and *NID1* or in their close neighborhood as strongly associated with the congenital cataract phenotype in Holstein calves with the *CPAMD8* wild type. In conclusion, the *CPAMD8*:g.5995966C>T variant is insufficient to explain the majority of Morgagnian congenital cataract phenotypes in Holsteins. It is very likely that congenital bilateral cataracts may be genetically heterogeneous and not yet known variants in genes other than *CPAMD8* and *NID1* are involved.

## Introduction

Unilateral or bilateral congenital cataracts with focal to diffuse opacities of the lens cause impaired vision to blindness in young cattle [1,2]. In cattle, cases of congenital cataracts were reported in many different breeds including Holstein [3–5], Swiss Brown [6], Jersey [7], Hereford [8], Aberdeen Angus [8], Shorthorn [9], Ayrshire [10] and Romagnola [11]. Prevalences were up to 34% in Holsteins [5], 79.5% [12] and 31% in a Canadian Holstein dairy herd [13]. Congenital nuclear cataracts are assumed to be due to impairment of early ocular organo-genesis [2] between 30 to 60 days of embryonic development [14]. Knowledge about etiologies of cataracts in cattle is still under debate [6, 11, 15], even inheritance seems to play a major role [1, 7] and genetic mutations have been proven in two different cattle breeds [11, 15]. An autosomal recessive inheritance was suggested for congenital cataracts in cattle [7], whereas in Jerseys an autosomal dominant condition was conclusive [1]. A deletion in the *NID1* gene (c.3579_3604+829del) was associated with a recessively inherited juvenile bilateral cataract in Romagnola cattle [11]. A nonsense mutation in the *CPAMD8* gene (g.5995966C>T) with a recessive mode of inheritance was perfectly associated with congenital Morgagnian cataracts in Red Holstein calves [15].

The objective of this study was to perform a validation study for the *CPAMD8*:g.5995966C>T variant in Holstein calves affected with congenital cataracts and in addition, for their unaffected relatives including dams, sires and paternal half-siblings as well as randomly selected herd mates. Calves with congenital cataracts underwent a clinical and in a few cases, a patho-histological examination was performed. In order to screen for further not yet known variants within candidate genes *CPAMD8* and *NID1* involved in bovine congenital cataracts, we analyzed whole genome sequencing (WGS) in three affected Holstein calves, two unaffected dams of affected calves and one unaffected herd mate.

## Materials and methods

### Ethics statement

All animal work was conducted according to the national and international guidelines for animal welfare. The present study was specifically approved by the Institutional Animal Care and Use Committee of Lower Saxony, the State Veterinary Office Niedersächsisches Landesamt für Verbraucherschutz und Lebensmittelsicherheit, Oldenburg, Germany (registration number 33.9-42502-05-04A247). Six calves were euthanized because of their unability for feed intake without support. The decision to euthanize the animals was at the discretion of the herd veterinarian. A written informed consent was obtained from cattle owners to use tissue samples for molecular genetic studies including WGS and data from their animals in the present study.

### Animals

In total, 396 EDTA blood or hair root samples from cattle were collected for the present study (S1 Table). Blood samples included 35 Holstein calves affected with congenital cataracts, whereof 27 cases were ascertained in the Clinic for Cattle, University of Veterinary Medicine Hannover, and eight cases in dairy farms. In two dairy farms (farm B and C) blood sampling from six animals was not possible. Related animals (sire, dam or herd mates) of the cases were sampled as far these animals were still alive and in addition, unaffected calves. We sampled affected Red Holstein calves (one case in the university clinic, cases from farm A and B) and cases with a common Red Holstein ancestor (farm C). Further 305 samples of unaffected animals were available as private controls including animals of eight different cattle breeds. Genomic DNA was isolated using standard ethanol fraction or chloroform fraction.

### Ophthalmic examination

All calves where the farmer noticed cloudiness of the eyes during the first week after birth underwent a complete ophthalmic examination including slit lamp biomicroscopy (SL-15, Kowa Japan) and ophthalmoscopy (PanOptic, Wellch Allyn USA). In addition, three female calves from farm A (case 1–3) and three calves, two male and one female (case 4–6) from farm C affected with bilateral congenital cataract were ophthalmologically examined using ultrasound scans of both eyes with MyLab^™^One VET (Esaote Europe, Maastricht, The Netherlands).

### Necropsy

A routine necropsy focusing on ocular pathology was performed on the three calves from farm C (case 4–6) with bilateral congenital cataract. Following macroscopic examination of both eyes, the right bulbus/lens was fixated for 24 hours in 10% buffered formalin and subsequently processed for histology. Histological lesions were evaluated on hematoxylin-eosin stained sections. In addition, bovine herpesvirus type 1 (BHV1) and bovine viral diarrhea virus (BVDV) was tested by immunofluorescence and culture, as well as RNA detection from bluetongue virus (BTV) using PCR.

### Pedigree analysis

Pedigree information for the affected animals and their relatives of farm A-C were obtained from owners of the animals. For cases sampled in the veterinary university clinic pedigree data were not available. The pedigree data of farm C were analyzed with the information of six affected calves, two females and four males, their dams and sires, three unaffected half-siblings and further one male calf related with the affected calves in the grandparent generation. We calculated relationship and inbreeding coefficients with data for six generations between affected calves and sires with OPTI-MATE version 4.0 [16]. In addition, contributions of ancestors to the inbreeding coefficients of affected calves were determined.

### Genotyping

We genotyped the nonsense mutation in the bovine *CPAMD8* gene (ENSBTAG00000009331) using a VIC and FAM-labeled TaqMan assay according to manufacturers’ guidelines (ABI7300 sequence detection system, Applied Biosystems, Life technologies, Darmstadt, Germany).

To genotype the animals for the deletion within the bovine *NID1* gene (ENSBTAG00000007244) using a polymerase chain reaction (PCR), we used the following combinations of primers [11]: forward primer 5´- CATCAGGGAAATCCTGCTGT-3´, first reverse primer (wild type) 5´- CAGGTGGGTTACCTTCAGGA-3´specific for a 176-bp amplicon and a second reverse primer (mutant) 5´- GTGACCTGGAAAAGGCAGAA-3´, which produces a 286-bp amplicon.

### Whole genome sequencing

For whole genome sequencing (WGS), genomic DNA of cases 4–6, the dam of case 4 and 5 as well as one unaffected herd mate from farm C was isolated using standard chloroform extraction. According to the manufacturers protocols libraries from these samples were prepared using the NEBNext Ultra DNA Library Prep Kit for Illumina (New England BioLabs, Ipswich, MA, USA). WGS was performed using the Illumina NextSeq500 (Illumina, San Diego, CA, USA) in a 2 x150 bp paired-end mode. Quality control for the data was performed using fastqc 0.11.5 [17] and the reads were trimmed using PRINSEQ, version 0.20.4 [18]. The resulting data were mapped to the bovine reference genome UMD 3.1 (Ensembl) using BWA 0.7.13 [19]. SAMtools 1.3.1 [20] and Picard tools (http://broadinstitute.github.io/picard/, version 2.9.4) were used for sorting, indexing and marking of duplicates of Bam-files. Variants were called with GATK, version 4.0 [21], using Base Quality Score Recalibration (BQSR), Haplotype Caller and Variant Recalibrator. We took all variants with a read depth of 2–999 and quality score values >20 were chosen from the vcf-file for further analysis.

These variant data of the six sequenced animals were compared with variant callings from 88 private controls of 12 different cattle breeds. All variants with high or moderate effects according to prediction toolbox SNPEff, version 4.3 q (2017-08-30, SNPEff database UMD3.1.86) [22] were selected.

The WGS data of the three affected calves were screened for mutant variants in the regions from 500 kb upstream to 500 kb downstream of *CPAMD8* (UMD3.1 Chr7:5995888–6095676 and ARS-UCD1.2 Chr7:6073318–6174087) and *NID1* (UMD3.1 Chr28:8733209–8829937 and ARS-UCD1.2 Chr28: 8687268–8784180) using SAS, version 9.4 (Statistical Analysis System, Cary, NC, USA). We filtered out variants homozygous mutant for the affected calves, heterozygous mutant for the dams, heterozygous mutant or wild type of the unaffected herd mate and homozygous wild type in the private controls.

Whole genome sequencing data of the six animals were deposited in NCBI Sequence Read Archive (http://www.ncbi.nlm.nih.gov/sra) under SRA accession number PRJNA526664 (SAMN11107031, SAMN11107033, SAMN11107034, SAMN11107035, SAMN11107036 and SAMN11107037).

### Structural variant detection

For screening chromosomal aberrations, we used the breakpoint prediction framework, LUMPY. We compared WGS data from cases 4–6 with data from two private control animals of the Holstein breed. Structural variants were filtered out which were either homozygous mutant in the affected calves and heterozygous or wild type in both controls or heterozygous mutant in the affected calves and wild type in both controls. Structural variants contained within or nearby to *CPAMD8* (UMD3.1 Chr7:5995888–6095676 and ARS-UCD1.2 Chr7:6073318–6174087) and *NID1* (UMD3.1 Chr28:8733209–8829937 and ARS-UCD1.2 Chr28: 8687268–8784180) were filtered out.

## Results

### Ophthalmic examination

**Fig 1:**
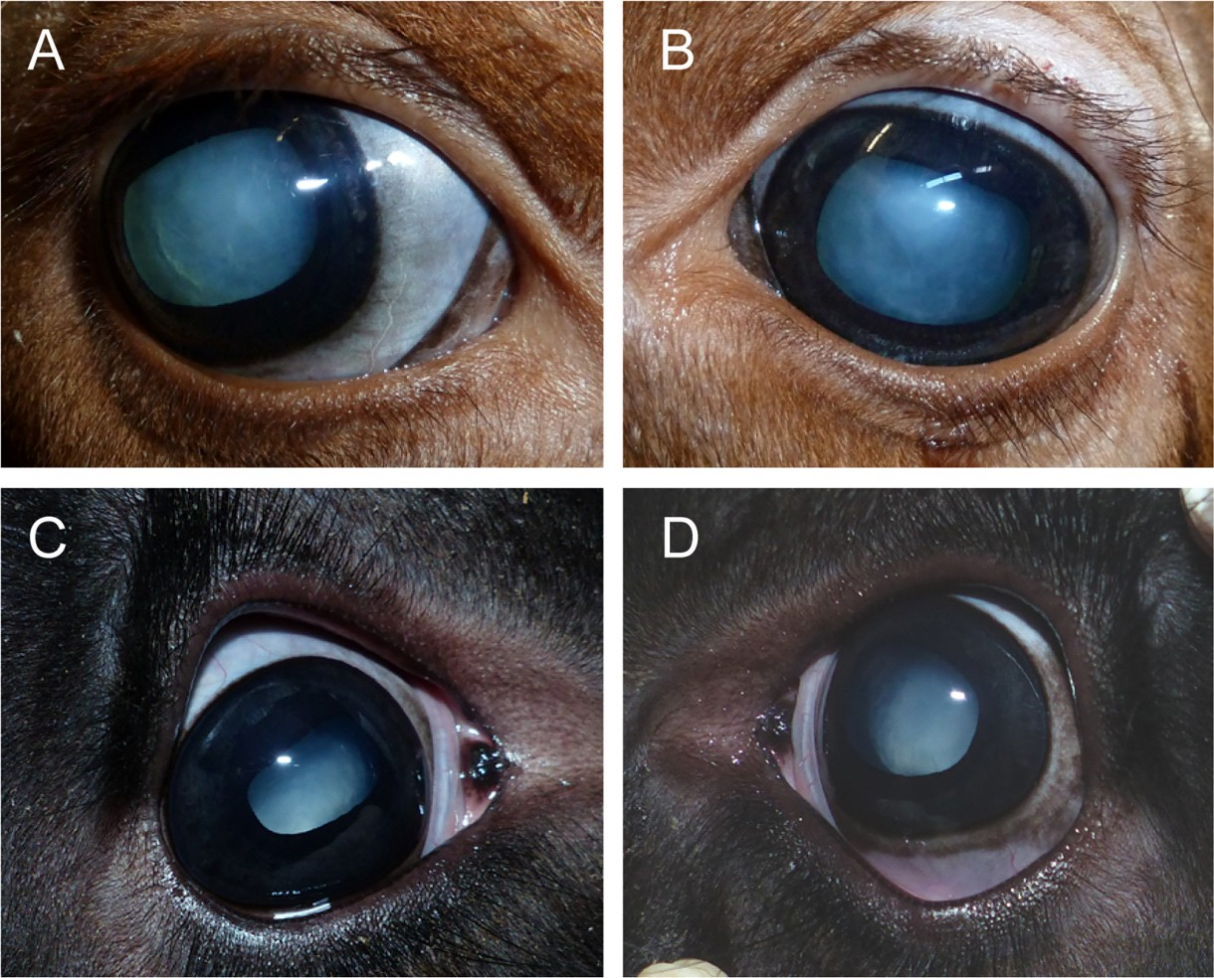
Bovine congenital bilateral cataract in Holstein calves. (A) The right and (B) the left eye of a Red Holstein calf from farm A with congenital opacities of the lens. The right (C) and the left eye (D) with opaque lenses of a polled Holstein calf from farm C are displayed.

The examination of all 41 affected calves revealed congenital bilateral cataract characterized by complete opacities of both lenses (Fig 1). No further abnormalities of the eyes were detected. For the six affected calves from farm A and C (cases 1–6) with ultrasound scans of both eyes, the detailed examination results of are summarized in Table 1. In all cases both eyes were open and showed no irritation with epiphora and no blepharospasm. The menace reflex was slightly positive in case 1, 2 and 4, almost completely negative in case 5 and 6 and negative in case 3. All calves reacted slightly positive of action of light. Pupillary reflexes of both eyes were negative in case 1, 2, 4, 5 and 6 and slightly positive in case 3. The irises of all calves were dark pigmented with a ventro-nasal iris coloboma in case 1 and 2. Besides complete opacities, bilateral microphakia was detected in the lens of case 1 and 2.

**Table 1:** Results of ophthalmological examinations.

Animal	Sex	Examination			Comments
		Menace reflex	Light reaction	Pupillary reflex	
**Farm A**					
Case 1	Female	+ r (+) l	+ r + l	- r - l	Both eyes open and non-irritant Iris dark pigmented, ventro-nasal iris coloboma Lens: microphakia, mature opacities Ultrasound scan: bilateral strands with vitreous Intraocular pressure: 18 mmHg (right), 20 mmHg (left)
Case 2	Female	+ r + l	+ r + l	- r - l	Both eyes open and non-irritant Iris dark pigmented, ventro-nasal iris coloboma Lens: microphakia, opacities Ultrasound scan: wispy hyperechogenic structures above the *N*. *opticus* Intraocular pressure: 18 mmHg (right), 16 mmHg (left)
Case 3	Female	- r - l	(+) r (+) l	+ r - l	Both eyes open and non-irritant Iris dark pigmented Lens: cortical and nucleus mature opacities Ultrasound scan: opacities Intraocular pressure: 18 mmHg (right), 16 mmHg (left)
**Farm C**					
Case 4	Male	+ r + l	+ r + l	(+) r (+) l	Both eyes open and non-irritant, slight epiphora Iris dark pigmented with extensive brightening Lens: microphakia, opacities Ultrasound scan: medium echogenic lens, lens diameter left 4.1x5 mm, right 2.4x4 mm
Case 5	Male	(+) r (+) l	(+) r (+) l	- r - l	Both eyes open and non-irritant Iris dark pigmented Lens: microphakia, opacities Ultrasound scan: incomplete (hypo-)/ medium echogenic lens, lens diameter left 6.4x8.5 mm, right 6x8.7 mm
Case 6	Female	(+) r (+) l	(+) r (+) l	- r - l	Both eyes open and non-irritant, no epiphora, no blepharospasm Iris dark pigmented Lens: microphakia, opacities Ultrasound scan: incomplete hypo-/medium echogenic lens, diameter of right and left lens 8x7 mm

Six with congenital bilateral cataract affected calves from farm A and C were ophthalmologically examined. The reactions of the eye reflexes for their right (r) and left (l) eyes are marked with the following symbols: + = slightly positive, ++ = moderately positive, +++ = highly positive,— = negative

The ultrasound scan displayed in case 1 bilateral strands with vitreous, wispy hyperechogenic structures above the *nervus opticus* in case 2 and opacities of the lens in case 3.

### Necropsy and patho-histological findings

**Fig 2:**
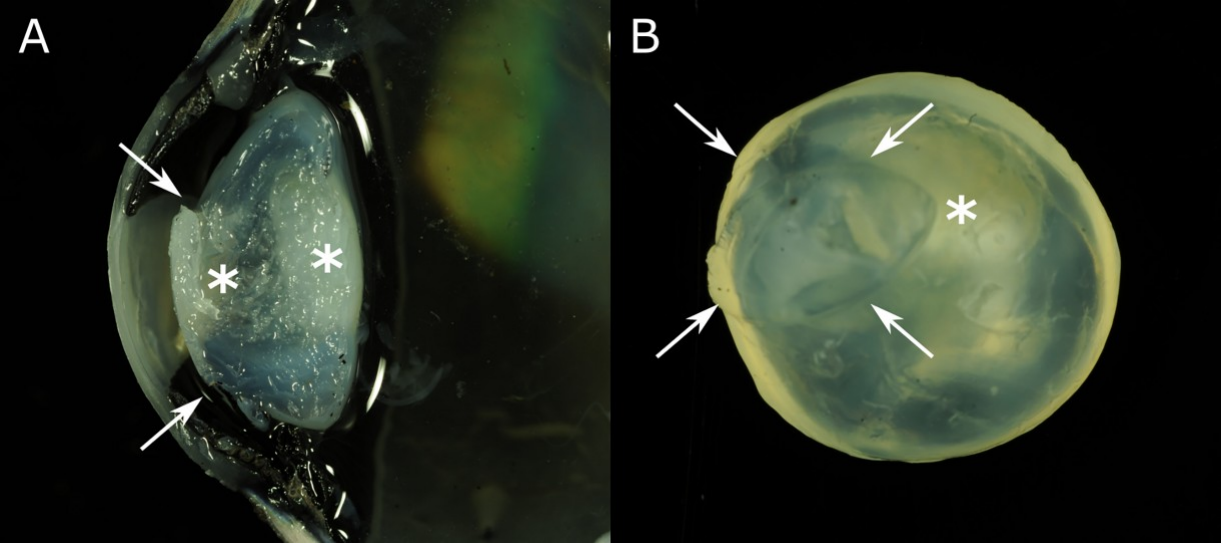
Gross findings in calves with congenital cataracts. (A) Cross section through the globe of case no. 5. (B) Anterior view of the isolated lens of case no. 6. Lenses show areas of turbidity in nuclear and cortical areas (asterisks). In addition, both lenses show a rupture of the anterior capsule with protrusion of lens material (areas delineated by arrows).

Macroscopically, the eyes of all three cases showed a microphakia with irregular lens contour and partial, nuclear to cortical opacity of the lens (Fig 2). Two calves showed an anterior rupture of the lens capsule with protrusion of lens material into the posterior chamber.

**Fig 3:**
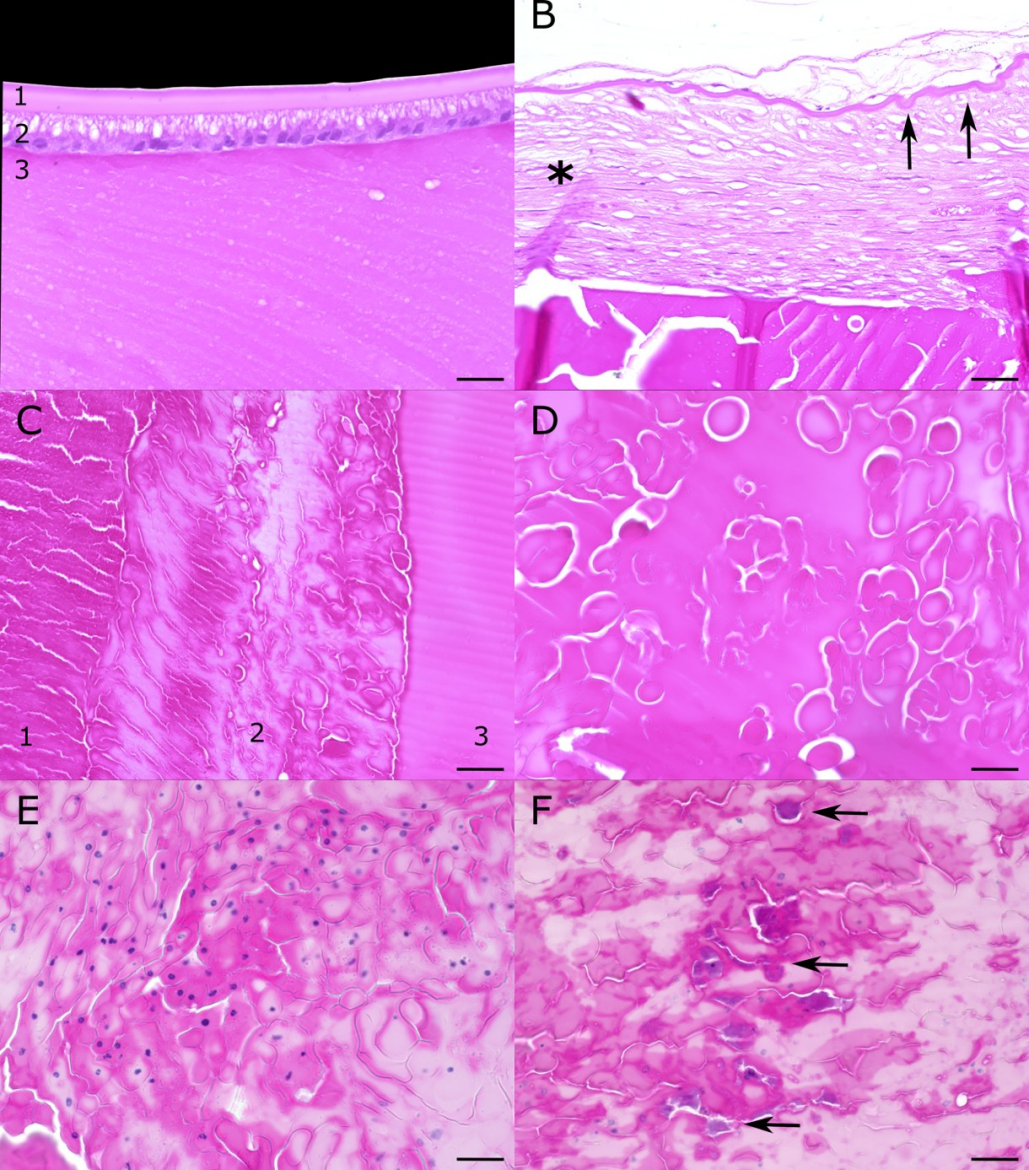
Histologic lesions in lenses from calves with congenital cataracts. Hematoxylin-eosin stain. Scale bars: A-C: 50 μm, D-F: 25 μm. (A) Intact anterior pole in a less affected calf (1) regular lens capsule (2) lens epithelium and (3) parallel lens fibers (case 4). (B) Posterior lens pole shows wrinkling of the capsule (arrow) and prominent subcapsular fibrosis (asterisk, case 5). (C) Gradual degeneration of lens fibers in the nucleus: (1) preserved fibers (2) fiber denaturation with formation of Morgagnian globules, (3) complete liquefaction of fibers into amorphous eosinophilic fluid (case 4). (D) Higher magnification of Morgagnian globules in the lens of (case 5). (E) Lenticular degeneration is accompanied by accumulation of bladder cells (case 6). (F) Dystrophic mineralization occurring in cataractous lesions (basophilic, granular material marked by arrows in case 6).

Microscopic examination of the right globe revealed nuclear to cortical cataract formation in all three cases, which varied in extent and severity (Table 2, Fig 3). Lenticular degeneration was characterized by fiber disarray, denaturation of fibers with formation of Morgagnian globules or complete liquefaction of lens material with accumulation of proteinaceous fluid. The degenerative lesions were accompanied by aggregates of bladder cells, which are presumed to represent abortive attempts at regeneration of lens fibers. Scattered foci of dystrophic mineralization were found in all three cases. These features are characteristic of hypermature cataract formation. In addition, two calves showed a mild to moderate subcapsular fibrosis and a wrinkled capsule at the posterior pole of the lens. In the most affected animals (case 5 and 6) the cataract extended from the posterior to the anterior pole and degenerated fiber material was protruding through a tear in the anterior capsule into the posterior eye chamber. In case 6, which also showed a rupture of the anterior capsule, the protruding lens material exhibited a regular morphology. No lesions were observed in other structures of the examined globes.

**Table 2:** Results of the patho-histological examination of lenses from calves with congenital cataracts.

Animal	Gross findings	Histology
**Case 4**	Microphakia Irregular lens contour Nuclear to posterior cortical opacity	Nuclear to posterior cortical and subcapsular cataract Central accumulation of eosinophilic amorphous material (liquefaction) with fiber disarray and Morgagnian globules Mineralization Posterior accumulations of bladder cells Posterior subcapsular fibrosis
**Case 5**	Microphakia Irregular lens contour Nuclear, anterior and posterior cortical opacity Rupture of anterior capsule with protrusion of opaque lens material	Nuclear, anterior and posterior cortical cataract Accumulation of eosinophilic amorphous material (liquefaction) with fiber disarray and formation of Morgagnian globules Mineralization Posterior accumulation of bladder cells Posterior subcapsular fibrosis and wrinkling of posterior capsule Rupture of anterior capsule with protrusion of degenerated lens protein
**Case 6**	Microphakia Irregular lens contour Nuclear opacity Rupture of anterior capsule	Nuclear cataract, partly extending into cortex Central accumulation of eosinophilic amorphous material (liquefaction) with fiber disarray and formation of Morgagnian globules Mineralization Accumulation of bladder cells Posterior subcapsular fibrosis and focal posterior subcapsular mineralization Rupture of anterior capsule with protrusion of lens material

The direct and indirect virus detections for BHV1, BVDV and BTV were negative.

### Pedigree analysis

**Fig 4:**
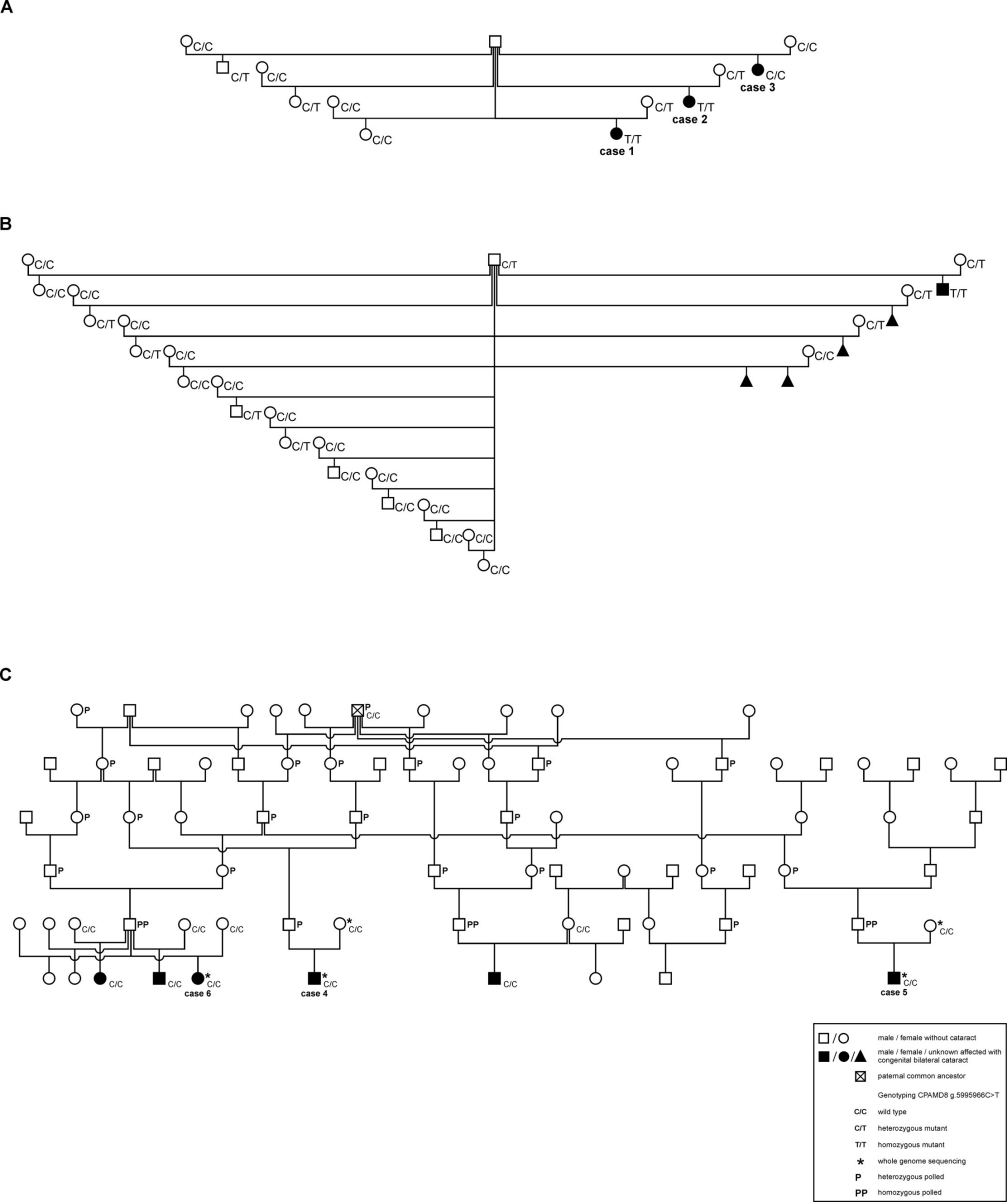
Pedigree analysis of animals from farm A, B and C. The affected calves showed congenital bilateral cataract. In farm C, all sires of affected calves were genetically polled insemination bulls. These sires could be traced back to a common ancestor, a heterozygous polled Red Holstein bull. Cases 1–6 were ophthalmologically and pathologically examined. Other cases underwent an ophthalmological examination. Asterisks mark animals used for whole genome sequencing.

Pedigrees of the affected calves from farm A, B and C could not be traced back to a common ancestor. However, only incomplete pedigree information was available from farm A and B (Fig 4). In the last offspring generation on farm C, 12 polled insemination sires were used for the first time, which had in total 27 offspring (13 female and 14 male). Calves of four polled sires were affected with congenital cataracts. In farm A and B each one farm owned sire was in use. The mean relationship coefficient between affected calves from farm A and B was 25% but no inbreeding was identified.

Analysis for common ancestors in farm C was based on a six generation pedigree. Pedigree completeness was 53.04% for the affected calves and 77.74% for the sires in this farm. Mean inbreeding coefficient for the affected calves was 0.59% with a range of 0.0–1.4%. A common ancestor contributing to inbreeding of the affected calves was not detected. Three Holstein bulls (O Man 007HO06417, Shottle 029HO12209 and Goldwin CAN10705608) mainly contributed to inbreeding coefficients of the affected calves. Relationship coefficients between affected calves were at 0.46 to 26.78% and a mean of 7.56%. The relationship coefficients between the different sires of the affected calves were between 2.22 and 9.98% with a mean of 4.95%. All sires included in the pedigree for cases on farm C were polled with a homozygous or heterozygous genotype and were traced back to a common ancestor, the heterozygous polled Red Holstein bull (Lawn Boy Red HO8223). The relationship coefficient of Lawn Boy Red and the sires of the affected calves amounted to 14.98% on average with a range of 9.23 to 26.59%. Between the dams no close relationship due to common ancestors was detected.

### Genotyping

In 3/35 genotyped affected calves, homozygous mutant genotypes of the *CPAMD8*:g.5995966C>T variant were validated (farm A: n = 2 and B: n = 1), whereas 32/35 affected calves showed the homozygous wild type (Table 3). These three homozygous *CPAMD8* mutated and affected calves were Red Holsteins. In all three farms, A, B and C, there were calves with the congenital cataract phenotype but the homozygous *CPAMD8* wild type. Each one affected calf from farm A and B were wild type. All affected calves from farm C and all cases from the Clinic for Cattle, University of Veterinary Medicine Hannover, were homozygous wild type.

**Table 3:** Genotyping results of *CPAMD8*:g.5995966C>T in 390 cattle and *NID1*:c.3579_3604+829del in 126 cattle.

Animals	Breed	Number of animals	*CPAMD8*:g.5995966C>T			*NID1*:c.3579_3604+829del		
			C/C	C/T	T/T	wt/wt	wt/del	del/del
Clinic for Cattle, University of Veterinary Medicine Hannover	Red Holstein	1	1	0	0	1	0	0
	Holstein	26	26	0	0	26	0	0
Farm A								
Affected calves Unaffected animals	Red Holstein	3	1	0	2	3	0	0
• dams of affected calves	Red Holstein	3	1	2	0	3	0	0
• dams of unaffected calves	Red Holstein	3	3	0	0	3	0	0
• paternal half siblings	Red Holstein	3	1	2	0	3		
Farm B								
Affected calf Unaffected animals	Red Holstein	1	0	0	1	1	0	0
• dams of affected calves	Red Holstein	4	1	3	0	4	0	0
• dams of unaffected calves	Holstein	10	10	0	0	10	0	0
• sire of affected calves	Red Holstein	1	0	1	0	1	0	0
• paternal half siblings	Holstein	10	6	4	0	-	-	-
• herd mates (cows)	Holstein	10	9	1	0	-	-	-
Farm C								
Affected calves Unaffected animals	Holstein	4	4	0	0	4	0	0
• dams of affected calves	Holstein	6	6	0	0	6	0	0
Controls								
	Holstein	94	94	0	0	6	0	0
	Polled Holstein	176	176	0	0	-	-	-
	Fleckvieh	5	5	0	0	5	0	0
	German Brown	5	5	0	0	5	0	0
	German Angus	5	5	0	0	5	0	0
	Limousin	5	5	0	0	5	0	0
	Charolais	5	5	0	0	5	0	0
	Salers	5	5	0	0	5	0	0
	Blonde d’Aquitaine	5	5	0	0	5	0	0
Total		390	282	14	3	126	0	0

The distributions of the wild type alleles (C, wt) and mutant alleles (T, del) are shown. Three affected Holstein calves were homozygous mutant for the variant *CPAMD8*:g.5995966C>T. The variant *NID1*:c.3579_3604+829del was wild type in all genotyped cattle.

Obligate heterozygous mutant genotypes were confirmed in 6/14 parents of affected calves from farm A, B and C. In farm A, two dams which gave birth to cataract affected calves, showed a heterozygous *CPAMD8* genotype as well as three dams and the sire of affected calves on farm B. In farm A, one dam with an affected calf was homozygous wild type. In farm B, the dam of two affected stillborn twins was homozygous wild type. All dams with affected calves from farm C were homozygous wild type.

Of the paternal half-siblings in farm B, 4/10 were heterozygous mutant as well as 1/10 unaffected herd mates. In summary, only in farm A and B, mutant alleles for the *CPAMD8*:g.5995966C>T variant were validated, while all other affected calves from farm C and all cases from the Clinic for Cattle, University of Veterinary Medicine Hannover, were homozygous wild type.

All samples tested for the Romagnola specific *NID1*:c.3579_3604+829del mutation were genotyped as homozygous wild type.

### Whole genome sequencing

Examination of filtered WGS data revealed in total 3563 variants within *CPAMD8* and *NID1* and 500 kb upstream and downstream to these genes in all examined animals, whereof 82/3563 were variants with moderate or high effects on protein function (S2 Table). None of these variants was private for the calves with congenital cataracts. The filtered variants were common in controls and the sequences of the 88 controls of the breeds Holstein, Fleckvieh, Braunvieh, Vorderwald, German Angus, Galloway, Limousin, Charolais, Hereford, Tyrolean Grey and Miniature Zebu. Structural variants were not found within *CPAMD8*. In addition, we were not able to identify affected calves as exclusively compound heterozygous for variants with moderate or high effects on protein function.

## Discussion

We confirmed the candidate variant *CPAMD8*:g.5995966C>T as familial consistent and associated with Morgagnian congenital cataract phenotypes in Red Holsteins but this does not necessarily imply that Red Holsteins or Holsteins with this condition are homozygous for the mutant allele. Segregation of this variant in Holsteins was also observed. The previously proposed simple Mendelian recessive inheritance of the cataract-associated variant was validated in two dairy farms in three affected calves genotyped as homozygous mutant and six obligate carriers. In addition, segregation was obvious in six unaffected paternal half-sibs and one herd mate from these two farms. But, for the majority of the examined cases, this mutation was not determined for congenital cataract phenotypes. Thus, only 3/35 cases were validated and the other cases remained unresolved. Our study indicated a very low frequency of this candidate variant in the German Holstein population, whereas congenital cataracts seem to be a wide-spread problem in Red Holstein [15], Holstein cattle [13] and also other cattle breeds [1–11]. Even if the pedigrees of all cases ascertained on three different farms included Red Holstein ancestors, we were not able to validate the candidate *CPAMD8* mutation.

Polled Holstein sires are now in wide use for artificial insemination. All these pedigrees also include Red Holstein ancestors. We screened 176 polled Holstein sires for the mutant *CPAMD8*:g.5995966C>T genotype, but were not able to find any heterozygous carrier.

Using whole genome sequencing, we could not identify variants in protein function affecting regions of the candidate genes *CPAMD8* and *NID1* which gave evidence for strong association of homozygous mutant genotypes with congenital cataracts in Holsteins from farm C. We did also not find private homozygous intronic variants in the regions of bovine candidate genes *CPAMD8* and *NID1* for these congenital cataract cases.

Therefore, we assume that the bovine congenital Morgagnian cataract may have its origin in more than one causative mutation. Mutations in moderate to high linkage disequilibrium forming a joint haplotype were ruled out for the candidate *CPAMD8*:g.5995966C>T variant. The candidate gene *NID1* did not segregate in Holsteins nor in any other cattle breeds genotyped here and thus, can be excluded for congenital cataracts in Holsteins which is in agreement with a previous study [11].

Clinical examination of all 41 affected calves revealed a mature congenital cataract which has most likely its origin in the embryonic lens development. The presence of Morgagnian globules in the lens of the examined calves from farm C confirmed the mature cataract and confirmed the previously reported *CPAMD8*-associated phenotype in Red Holsteins [15]. Clinical signs among affected calves did not differ between homozygous *CPAMD8* mutant and wild type individuals. Histopathological findings in our cases 4–6 were very similar to previously reported Red Holstein calves homozygous *CPAMD8* mutant [15]. Thus, morphological findings are not supportive for a specific genetic mutation or an environmental cause.

A possible infectious etiology due to BVDV for bovine congenital cataracts [23] has to be taken into account as a possible causative agent besides inherited forms. Infection with BVDV between approximately 75 to 150 days of gestation may result in congenital malformations including equatorial and subcapsular cataracts, uveitis, retinal degeneration and optic neuritis [23]. These animals are born antibody negative but BVDV positive [24]. Infections later in gestation were not associated with varying degrees of blindness in newborn calves [23, 24]. All animals used for this study and their herd mates from these dairy farms were tested free from BVDV. Tissues of cases 4–6 obtained at necropsy, tested negative for BVDV, BHV1 and BTV. Therefore, an infection with BVDV seems rather unlikely as the cause of the congenital cataracts of the animals under study here. This is in agreement with a previous study in Red Holsteins [15].

Farmers are faced with animal welfare issues to keep nearly blind or blind calves in their herd because daily feeding and handling is labor intensive and animals with impaired vision are low ranking and may experience aggression from other herd mates [25].

In conclusion, our validation study of the *CPAMD8*:g.5995966C>T variant showed the presence of this mutation in a very low frequency in cases with a confirmed diagnosis of congenital cataract in Holstein calves. A systematic screening program for breeding animals for the *CPAMD8*:g.5995966C>T variant will not be very effective to reduce congenital cataracts due to the very low sensitivity of the *CPAMD8* candidate mutation to detect unaffected carriers. Using whole genome sequencing data from calves with congenital cataracts and wild type for *CPAMD8*:g.5995966C>T did not reveal variants strongly associated with this phenotype. Further studies are needed to uncover candidate variants for congenital cataracts in Holsteins. Congenital cataract cases cause animal welfare issues due to missing appropriate handling of these animals.

## Supporting information

S1 Table Distribution of congenital cataract phenotypes and unaffected controls as well as samples collected for genotyping. (DOCX)

S2 Table Filtering results of whole genome sequencing data revealed 83 protein function affecting variants in the candidate genes *CPAMD8* and *NID1* genes as well as 500 kb upstream and downstream of these genes. None of these variants was associated with the congenital cataract phenotype exclusively present in the affected calves in an autosomal recessive mode of inheritance. These variants were also found in 88 private controls of the breeds Holstein, Fleckvieh, Braunvieh, Vorderwald, German Angus, Galloway, Limousin, Charolais, Hereford, Tyrolean Grey and Miniature Zebu.
(DOCX)
