# Supplementary material for: Study of congenital Morgagnian cataracts in Holstein calves
Source: PLoS One. 2019 Dec 26;14(12):e0226823. doi: 10.1371/journal.pone.0226823 (PMC6932804; doi:10.1371/journal.pone.0226823)
Supplement: S1 Table — (DOCX) [file pone.0226823.s001.docx]

**Table S1.** Distribution of congenital cataract phenotypes and unaffected controls as well as samples collected for genotyping.

| **Animals** | | **Breed** | **Ascertained phenotype** | | **Number of samples** | |
| --- | --- | --- | --- | --- | --- | --- |
|  |  |  | **Congenital cataract** | **Unaffected controls** | **Congenital cataract** | **Unaffected controls** |
| Veterinary university clinic | | Red Holstein | 1 | 0 | 1 | 0 |
|  | | Holstein | 26 | 0 | 26 | 0 |
| Farm A | |  |  |  |  |  |
| Calves | | Red Holstein | 3 | 0 | 3 | 0 |
| Dams | | Red Holstein | 0 | 6 | 0 | 6 |
| Paternal half-sibs (calves) | | Red Holstein | 0 | 3 | 0 | 3 |
| Farm B | |  |  |  |  |  |
| Calves | | Holstein | 5 | 10 | 1 | 10 |
| Dams | | Holstein | 0 | 14 | 0 | 14 |
| Sires | | Red Holstein | 0 | 1 | 0 | 1 |
| Herdmates | | Holstein | 0 | 10 | 0 | 10 |
| Farm C | |  |  |  |  |  |
| Calves | | Holstein | 6 | 0 | 4 | 0 |
| Dams | | Holstein | 0 | 6 | 0 | 6 |
| Controls | |  |  |  |  |  |
|  | | Holstein | 0 | 94 | 0 | 94 |
|  | | Polled Holstein | 0 | 176 | 0 | 176 |
|  | | Fleckvieh | 0 | 5 | 0 | 5 |
|  | | German Brown | 0 | 5 | 0 | 5 |
|  | | German Angus | 0 | 5 | 0 | 5 |
|  | | Limousin | 0 | 5 | 0 | 5 |
|  | | Charolais | 0 | 5 | 0 | 5 |
|  | | Salers | 0 | 5 | 0 | 5 |
|  | | Blonde d’Aquitaine | 0 | 5 | 0 | 5 |
| Total |  | | 41 | 355 | 35 | 355 |
