# Supplementary material for: Study of congenital Morgagnian cataracts in Holstein calves
Source: PLoS One. 2019 Dec 26;14(12):e0226823. doi: 10.1371/journal.pone.0226823 (PMC6932804; doi:10.1371/journal.pone.0226823)
Supplement: S2 Table — None of these variants was associated with the congenital cataract phenotype exclusively present in the affected calves in an autosomal recessive mode of inheritance. These variants were also found in 88 private controls of the breeds Holstein, Fleckvieh, Braunvieh, Vorderwald, German Angus, Galloway, Limousin, Charolais, Hereford, Tyrolean Grey and Miniature Zebu. (DOCX) [file pone.0226823.s002.docx]

**Table S2.** Filtering results of whole genome sequencing data revealed 83 protein function affecting variants in the candidate genes *CPAMD8* and *NID1* genes as well as 500 kb upstream and downstream of these genes. None of these variants was associated with the congenital cataract phenotype exclusively present in the affected calves in an autosomal recessive mode of inheritance. These variants were also found in 88 private controls of the breeds Holstein, Fleckvieh, Braunvieh, Vorderwald, German Angus, Galloway, Limousin, Charolais, Hereford, Tyrolean Grey and Miniature Zebu.

| **Gene** | **BTA** | **Position** | **ID** | **Ref** | **Alt** | **Case 4** | **Case 5** | **Case 6** | **Dam 1** | **Dam 2** | **Herd mate** | **Transcript** | **cDNA** | **Protein** |
| --- | --- | --- | --- | --- | --- | --- | --- | --- | --- | --- | --- | --- | --- | --- |
| ENSBTAG00000025767 | 7 | 5602912 | rs43497544 | T | C | 1/1 | 1/1 | 1/1 | 1/1 | 1/1 | 1/1 | ENSBTAT00000047511.3 | c.589T>C | p.Cys197Arg |
| ENSBTAG00000045588 | 7 | 5655211 | rs110389679 | A | G | 0/1 | 0/1 | 0/1 | 0/1 | 0/1 | 0/1 | ENSBTAT00000066038.1 | c.87A>G | p.Lys29Lys |
| ENSBTAG00000045588 | 7 | 5655246 | rs110150525 | T | C | 1/1 | 1/1 | 1/1 | 1/1 | 1/1 | 1/1 | ENSBTAT00000066038.1 | c.122T>C | p.Val41Ala |
| ENSBTAG00000045588 | 7 | 5655253 | rs209046319 | G | A | 0/1 | 0/1 | 0/1 | 0/1 | 0/1 | 0/1 | ENSBTAT00000066038.1 | c.129G>A | p.Val43Val |
| ENSBTAG00000045588 | 7 | 5655254 | rs457140911 | G | A | 0/1 | 0/1 | 0/1 | 0/1 | 0/1 | 0/1 | ENSBTAT00000066038.1 | c.130G>A | p.Gly44Arg |
| ENSBTAG00000045588 | 7 | 5655256 | rs210623953 | G | T | 0/1 | 0/1 | 0/1 | 0/1 | 0/1 | 0/1 | ENSBTAT00000066038.1 | c.132G>T | p.Gly44Gly |
| ENSBTAG00000045588 | 7 | 5655264 | rs208265697 | T | G | 0/1 | 0/1 | 0/1 | 0/1 | 0/1 | 0/1 | ENSBTAT00000066038.1 | c.140T>G | p.Val47Gly |
| ENSBTAG00000045588 | 7 | 5655266 | rs210329113 | C | A | 0/1 | 0/1 | 0/1 | 0/1 | 0/1 | 0/1 | ENSBTAT00000066038.1 | c.142C>A | p.Pro48Thr |
| ENSBTAG00000045588 | 7 | 5655269 | rs383526025 | A | C | 0/1 | 0/1 | 0/1 | 0/1 | 0/1 | 0/1 | ENSBTAT00000066038.1 | c.145A>C | p.Met49Leu |
| ENSBTAG00000045588 | 7 | 5655334 | rs210677353 | G | A | 0/1 | 0/1 | 0/1 | 0/1 | 0/1 | 0/1 | ENSBTAT00000066038.1 | c.210G>A | p.Glu70Glu |
| ENSBTAG00000045588 | 7 | 5655392 | rs210339532 | A | C | 0/1 | 0/1 | 0/1 | 0/1 | 0/1 | 0/1 | ENSBTAT00000066038.1 | c.268A>C | p.Asn90His |
| ENSBTAG00000045588 | 7 | 5655395 | rs211567585 | G | A | 0/1 | 0/1 | 0/1 | 0/1 | 0/1 | 0/1 | ENSBTAT00000066038.1 | c.271G>A | p.Glu91Lys |
| ENSBTAG00000045588 | 7 | 5655401 | rs109807009 | A | G | 0/1 | 0/1 | 0/1 | 0/1 | 0/1 | 0/1 | ENSBTAT00000066038.1 | c.277A>G | p.Lys93Glu |
| ENSBTAG00000045588 | 7 | 5655406 | rs109421834 | C | T | 0/1 | 0/1 | 0/1 | 0/1 | 0/1 | 0/1 | ENSBTAT00000066038.1 | c.282C>T | p.Ala94Ala |
| ENSBTAG00000045588 | 7 | 5656252 | rs133619471 | A | G | 1/1 | 1/1 | 1/1 | 1/1 | 1/1 | 1/1 | ENSBTAT00000066038.1 | c.363A>G | p.Gln121Gln |
| ENSBTAG00000045588 | 7 | 5656538 | rs379234434 | G | T | 1/1 | 0/1 | 0/1 | 0/1 | 0/1 | 0/1 | ENSBTAT00000066038.1 | c.367G>T | p.Glu123* |
| ENSBTAG00000045588 | 7 | 5656540 | rs475316802 | G | A | 0/1 | 0/1 | 0/1 | 0/0 | 0/1 | 0/1 | ENSBTAT00000066038.1 | c.369G>A | p.Glu123Glu |
| ENSBTAG00000045588 | 7 | 5656548 | rs432982809 | C | G | 0/1 | 0/1 | 0/1 | 0/0 | 0/1 | 0/1 | ENSBTAT00000066038.1 | c.377C>G | p.Thr126Ser |
| ENSBTAG00000045588 | 7 | 5656549 | rs444064483 | C | T | 0/1 | 0/1 | 0/1 | 0/0 | 0/1 | 0/1 | ENSBTAT00000066038.1 | c.378C>T | p.Thr126Thr |
| ENSBTAG00000045588 | 7 | 5656567 | rs454600777 | T | A | 0/1 | 0/1 | 0/1 | 0/0 | 0/1 | 0/1 | ENSBTAT00000066038.1 | c.396T>A | p.His132Gln |
| **Gene** | **BTA** | **Position** | **ID** | **Ref** | **Alt** | **Case 4** | **Case 5** | **Case 6** | **Dam 1** | **Dam 2** | **Herd mate** | **Transcript** | **cDNA** | **Protein** |
| ENSBTAG00000045588 | 7 | 5656574 | rs521355873 | T | TGA | 0/1 | 0/1 | 0/1 | 0/1 | 0/1 | 0/1 | ENSBTAT00000066038.1 | c.403_404  insGA | p.Ser135fs |
| ENSBTAG00000045588 | 7 | 5656577 | rs474073299 | G | A | 0/1 | 0/1 | 0/1 | 0/1 | 0/1 | 0/1 | ENSBTAT00000066038.1 | c.406G>A | p.Glu136Lys |
| ENSBTAG00000045588 | 7 | 5656861 | rs440765572 | T | C | 0/1 | 0/1 | 0/1 | 0/1 | 0/1 | 0/1 | ENSBTAT00000066038.1 | c.432T>C | p.Ala144Ala |
| ENSBTAG00000045588 | 7 | 5656864 | rs207540611 | A | C | 0/1 | 1/1 | 0/1 | 0/1 | 0/1 | 0/1 | ENSBTAT00000066038.1 | c.435A>C | p.Ser145Ser |
| ENSBTAG00000045588 | 7 | 5656867 | rs440188681 | A | G | 0/1 | 0/1 | 0/1 | 0/1 | 0/1 | 0/1 | ENSBTAT00000066038.1 | c.438A>G | p.Ala146Ala |
| ENSBTAG00000045588 | 7 | 5656874 |  | T | A,* | 0/2 | 0/1 | 0/1 | 0/1 | 0/1 | 0/1 | ENSBTAT00000066038.1 | c.445T>A | p.Tyr149Asn |
| ENSBTAG00000045588 | 7 | 5656885 |  | A | G,* | 0/2 | 0/1 | 0/1 | 0/1 | 0/1 | 0/1 | ENSBTAT00000066038.1 | c.456A>G | p.Arg152Arg |
| ENSBTAG00000045588 | 7 | 5656888 |  | C | CAG,* | 0/2 | 0/1 | 0/1 | 0/1 | 0/1 | 0/1 | ENSBTAT00000066038.1 | c.459_460  insAG | p.Val155fs |
| ENSBTAG00000045588 | 7 | 5656941 |  | T | TAC | 0/1 | 0/1 | 0/1 | 0/1 | 0/1 | 0/1 | ENSBTAT00000066038.1 | c.512_513  insAC | p.Leu172fs |
| ENSBTAG00000045588 | 7 | 5656950 | rs453842530 | C | T | 0/1 | 0/1 | 0/1 | 0/1 | 0/1 | 0/1 | ENSBTAT00000066038.1 | c.521C>T | p.Thr174Ile |
| ENSBTAG00000045588 | 7 | 5656957 | rs209073697 | A | G | 1/1 | 1/1 | 1/1 | 1/1 | 1/1 | 1/1 | ENSBTAT00000066038.1 | c.528A>G | p.Lys176Lys |
| ENSBTAG00000045588 | 7 | 5656991 | rs385121486 | A | G | 1/1 | 1/1 | 1/1 | 1/1 | 1/1 | 1/1 | ENSBTAT00000066038.1 | c.*19A>G |  |
| ENSBTAG00000045588 | 7 | 5656995 | rs137105520 | T | C,A | 1/1 | 1/1 | 1/2 | 1/1 | 2/2 | 1/2 | ENSBTAT00000066038.1 | c.*19+4T>A |  |
| ENSBTAG00000008021 | 7 | 5668778 | rs43498340 | G | A | 1/1 | 1/1 | 1/1 | 1/1 | 1/1 | 1/1 | ENSBTAT00000010544.5 | c.37G>A | p.Asp13Asn |
| ENSBTAG00000008021 | 7 | 5668811 | rs110547930 | C | T | 1/1 | 1/1 | 1/1 | 0/1 | 1/1 | ./. | ENSBTAT00000010544.5 | c.70C>T | p.Pro24Ser |
| ENSBTAG00000008021 | 7 | 5668832 | rs134883284 | T | TGTGACCG  CAAGCTGC  CGCTGG,G | 1/1 | ./. | 1/1 | 1/1 | 1/1 | 1/1 | ENSBTAT00000010544.5 | c.91T>G | p.Trp31Gly |
| ENSBTAG00000008021 | 7 | 5668838 | rs210864471 | C | G | 1/1 | ./. | 1/1 | 1/1 | 1/1 | 1/1 | ENSBTAT00000010544.5 | c.97C>G | p.Arg33Gly |
| ENSBTAG00000008021 | 7 | 5668892 | rs43498338 | G | A | 1/1 | 1/1 | 1/1 | 1/1 | 1/1 | 1/1 | ENSBTAT00000010544.5 | c.151G>A | p.Ala51Thr |
| ENSBTAG00000008021 | 7 | 5668899 | rs43498337 | T | C | 1/1 | 1/1 | 1/1 | 1/1 | 1/1 | 1/1 | ENSBTAT00000010544.5 | c.158T>C | p.Ile53Thr |
| ENSBTAG00000008021 | 7 | 5670011 | rs43498334 | A | G | 1/1 | 1/1 | 1/1 | 1/1 | 1/1 | 1/1 | ENSBTAT00000010544.5 | c.333A>G | p.Glu111Glu |
| ENSBTAG00000008021 | 7 | 5670015 | rs43498333 | A | G | 1/1 | 1/1 | 1/1 | 1/1 | 1/1 | 1/1 | ENSBTAT00000010544.5 | c.337A>G | p.Thr113Ala |
| ENSBTAG00000008021 | 7 | 5670300 | rs109508546 | G | C | 1/1 | 1/1 | 0/1 | 0/1 | 1/1 | 0/1 | ENSBTAT00000010544.5 | c.366G>C | p.Leu122Phe |
| ENSBTAG00000008021 | 7 | 5670647 | rs211435706 | C | T | 1/1 | 1/1 | 1/1 | 1/1 | 1/1 | 1/1 | ENSBTAT00000010544.5 | c.440C>T | p.Ser147Phe |
| ENSBTAG00000008021 | 7 | 5670723 | rs109404320 | C | A | 1/1 | 1/1 | 1/1 | 1/1 | 1/1 | 1/1 | ENSBTAT00000010544.5 | c.516C>A | p.Thr172Thr |
| PLVAP | 7 | 5698069 | rs109434229 | T | C | 1/1 | 1/1 | 1/1 | 1/1 | 1/1 | 1/1 | ENSBTAT00000052388.2 | c.1180-3T>C |  |
| **Gene** | **BTA** | **Position** | **ID** | **Ref** | **Alt** | **Case 4** | **Case 5** | **Case 6** | **Dam 1** | **Dam 2** | **Herd mate** | **Transcript** | **cDNA** | **Protein** |
| PLVAP | 7 | 5698122 | rs110957064 | T | A | 1/1 | 1/1 | 1/1 | 1/1 | 1/1 | 1/1 | ENSBTAT00000052388.2 | c.1230T>A | p.Pro410Pro |
| ABHD8 | 7 | 5749046 | rs133235871 | G | T | 0/1 | 0/1 | 1/1 | 0/1 | 1/1 | 1/1 | ENSBTAT00000036501.4 | c.795G>T | p.Val265Val |
| USHBP1 | 7 | 5769742 | rs109701849 | G | A | 1/1 | 0/1 | 0/1 | 0/1 | 0/1 | 0/0 | ENSBTAT00000020104.5 | c.476G>A | p.Gly159Glu |
| USHBP1 | 7 | 5774810 | rs43498404 | T | C | 1/1 | 0/1 | 0/1 | 0/1 | 0/1 | 0/0 | ENSBTAT00000020104.5 | c.1314T>C | p.Ser438Ser |
| MYO9B | 7 | 5836326 | rs43499541 | T | G | 1/1 | 1/1 | 1/1 | 1/1 | 1/1 | 1/1 | ENSBTAT00000014779.3 | c.2125A>C | p.Arg709Arg |
| HAUS8 | 7 | 5929224 | rs483329889 | TA | T | 1/1 | 1/1 | 1/1 | 1/1 | 1/1 | 1/1 | ENSBTAT00000047333.2 | c.160delA | p.Thr54fs |
| CPAMD8 | 7 | 6013286 | rs208854828 | A | G | 0/1 | 0/1 | 1/1 | 1/1 | 1/1 | 1/1 | ENSBTAT00000047290.2 | c.634A>G | p.Ser212Gly |
| CPAMD8 | 7 | 6016069 | rs137119279 | A | G | 0/1 | 0/1 | 0/1 | 1/1 | 1/1 | 1/1 | ENSBTAT00000047290.2 | c.796A>G | p.Ile266Val |
| CPAMD8 | 7 | 6084957 | rs110710592 | T | C | 0/1 | 0/1 | 0/1 | 0/0 | 1/1 | 0/1 | ENSBTAT00000047290.2 | c.4488T>C | p.Pro1496Pro |
| CPAMD8 | 7 | 6089431 | rs43501785 | T | C | 1/1 | 0/1 | 1/1 | 0/1 | 1/1 | 0/1 | ENSBTAT00000047290.2 | c.4919-5T>C |  |
| NWD1 | 7 | 6162022 | rs134932801 | C | T | 0/1 | 0/1 | 1/1 | 0/0 | 0/1 | 0/1 | ENSBTAT00000012281.5 | c.3901G>A | p.Gly1301Arg |
| NWD1 | 7 | 6163772 | rs43500002 | T | C | 0/1 | 1/1 | 0/1 | 0/1 | 0/1 | 1/1 | ENSBTAT00000012281.5 | c.3711A>G | p.Lys1237Lys |
| NWD1 | 7 | 6223973 | rs43499319 | G | T | 1/1 | 1/1 | 0/1 | 1/1 | 1/1 | 1/1 | ENSBTAT00000012281.5 | c.46C>A | p.His16Asn |
| ENSBTAG00000002447 | 7 | 6434821 | rs109772235 | T | C | 1/1 | 0/1 | 1/1 | 0/1 | 1/1 | 0/1 | ENSBTAT00000003176.4 | c.421A>G | p.Met141Val |
| ENSBTAG00000027878 | 7 | 6445836 | rs43506209 | A | G | 1/1 | 0/1 | 1/1 | 0/1 | 1/1 | 0/1 | ENSBTAT00000024473.5 | c.36T>C | p.Val12Val |
| CALR3 | 7 | 6466575 | rs43504859 | A | G | 1/1 | 1/1 | 1/1 | 1/1 | 1/1 | 1/1 | ENSBTAT00000013175.5 | c.436A>G | p.Ile146Val |
| EPS15L1 | 7 | 6532257 | rs135666502 | G | A | 0/1 | 1/1 | 0/1 | 1/1 | 0/1 | 1/1 | ENSBTAT00000017060.5 | c.822G>A | p.Ala274Ala |
| EPS15L1 | 7 | 6552107 | rs43504831 | T | C | 1/1 | 1/1 | 0/1 | 1/1 | 0/1 | 1/1 | ENSBTAT00000017060.5 | c.1792-3T>C |  |
| EPS15L1 | 7 | 6589608 | rs109280539 | T | C | 0/1 | 1/1 | 0/1 | 0/1 | 0/1 | 1/1 | ENSBTAT00000017060.5 | c.2388T>C | p.Ala796Ala |
| GNG4 | 28 | 8377879 | rs109880232 | A | G | 1/1 | 0/1 | 0/1 | 0/0 | 0/1 | 1/1 | ENSBTAT00000003777.2 | c.99+6T>C |  |
| LYST | 28 | 8424588 | rs136241371 | TA | T | 1/1 | 1/1 | 1/1 | 1/1 | 1/1 | 1/1 | ENSBTAT00000022355.5 | c.11253-6delT |  |
| LYST | 28 | 8450923 | rs211301704 | G | A | 1/1 | 1/1 | 1/1 | 1/1 | 1/1 | 1/1 | ENSBTAT00000022355.5 | c.9936C>T | p.Asn3312Asn |
| LYST | 28 | 8465283 | rs207693195 | T | C | 0/1 | 0/1 | 1/1 | 0/1 | 0/1 | 0/1 | ENSBTAT00000022355.5 | c.9315A>G | p.Val3105Val |
| LYST | 28 | 8492389 | rs208276890 | G | A | 1/1 | 1/1 | 1/1 | 1/1 | 1/1 | 1/1 | ENSBTAT00000022355.5 | c.7724C>T | p.Ala2575Val |
| NID1 | 28 | 8733559 | rs137783167 | G | A | 1/1 | 0/1 | 1/1 | 0/0 | 0/1 | 1/1 | ENSBTAT00000009531.5 | c.3699C>T | p.Ile1233Ile |
| NID1 | 28 | 8733580 | rs110406081 | G | A | 1/1 | 0/1 | 1/1 | 0/0 | 0/1 | 1/1 | ENSBTAT00000009531.5 | c.3678C>T | p.Asn1226Asn |
| NID1 | 28 | 8736122 | rs209300927 | C | T | 0/1 | 0/1 | 1/1 | 0/0 | 1/1 | 0/0 | ENSBTAT00000009531.5 | c.3362G>A | p.Arg1121Gln |
| **Gene** | **BTA** | **Position** | **ID** | **Ref** | **Alt** | **Case 4** | **Case 5** | **Case 6** | **Dam 1** | **Dam 2** | **Herd mate** | **Transcript** | **cDNA** | **Protein** |
| NID1 | 28 | 8798180 | rs208559281 | T | C | 1/1 | 1/1 | 1/1 | 1/1 | 1/1 | 1/1 | ENSBTAT00000009531.5 | c.640A>G | p.Thr214Ala |
| NID1 | 28 | 8798200 | rs210307350 | A | G | 1/1 | 1/1 | 1/1 | 1/1 | 1/1 | 1/1 | ENSBTAT00000009531.5 | c.620T>C | p.Phe207Ser |
| NID1 | 28 | 8798242 | rs210815305 | A | G | 1/1 | 1/1 | 1/1 | 1/1 | 1/1 | 1/1 | ENSBTAT00000009531.5 | c.578T>C | p.Leu193Ser |
| NID1 | 28 | 8798266 | rs384557059 | G | A | 0/1 | 1/1 | 1/1 | 0/1 | 1/1 | 0/1 | ENSBTAT00000009531.5 | c.554C>T | p.Thr185Met |
| ERO1B | 28 | 9026323 | rs380451978 | A | C | 1/1 | 1/1 | 1/1 | 1/1 | 1/1 | 1/1 | ENSBTAT00000001099.5 | c.28T>G | p.Ser10Ala |
| HEATR1 | 28 | 9249810 | rs109898608 | T | C | 1/1 | 0/1 | 1/1 | 1/1 | 1/1 | 0/1 | ENSBTAT00000061542.2 | c.5001A>G | p.Thr1667Thr |
| HEATR1 | 28 | 9250434 | rs208286050 | G | C | 1/1 | 1/1 | 1/1 | 1/1 | 1/1 | 1/1 | ENSBTAT00000061542.2 | c.4875C>G | p.Asn1625Lys |
| HEATR1 | 28 | 9253259 | rs135527035 | G | C | 1/1 | 0/1 | 1/1 | 0/1 | 1/1 | 0/1 | ENSBTAT00000061542.2 | c.4596+3C>G |  |
| HEATR1 | 28 | 9255542 | rs109561809 | G | A | 0/1 | 0/1 | 1/1 | 0/1 | 1/1 | 0/1 | ENSBTAT00000061542.2 | c.4197C>T | p.Leu1399Leu |
| HEATR1 | 28 | 9255584 | rs135982154 | A | G | 0/1 | 0/1 | 1/1 | 0/1 | 1/1 | 0/1 | ENSBTAT00000061542.2 | c.4155T>C | p.Asp1385Asp |
| HEATR1 | 28 | 9273258 | rs110499005 | T | C | 1/1 | 1/1 | 1/1 | 1/1 | 1/1 | 1/1 | ENSBTAT00000061542.2 | c.1767A>G | p.Leu589Leu |
